# Could diet and body composition influence vaginitis treatment success? A clinical nutrition-based analysis

**DOI:** 10.3389/fnut.2026.1882788

**Published:** 2026-08-05

**Authors:** Aybala Tazeoglu, Yasemin Ergul

**Affiliations:** 1 Department of Nutrition and Dietetics, Faculty of Health Sciences, Osmaniye Korkut Ata University, Osmaniye, Türkiye; 2 Department of Nutrition and Dietetics, Faculty of Health Sciences, Bandirma Onyedi Eylul University, Bandirma, Türkiye

**Keywords:** abdominal obesity, diabetes mellitus, dietary carbohydrate, fiber intake, treatment failure, vaginal delivery, vaginitis

## Abstract

**Purpose:**

To investigate the association between anthropometric characteristics, dietary composition, and obstetric/gynecological history and the risk of treatment failure among women diagnosed with vaginitis.

**Methods:**

A total of 106 women diagnosed with vaginitis were included in this prospective observational study. Patients were divided into two groups: those who recovered after treatment and those with treatment-resistant infections. Demographic data, body mass index (BMI), abdominal fat ratio, daily fiber intake, dietary carbohydrate and simple sugar percentages, diabetes status, menopausal state, Obstetric history, and prior episodes of vaginitis were recorded. Comparisons between groups were analyzed using *t*-tests and Chi-square tests. Multivariate logistic regression was used to identify predictors of treatment failure.

**Results:**

Patients in the treatment-resistant group had significantly higher BMI, abdominal fat ratio, and dietary carbohydrate and simple sugar intake (*p* < 0.05). Fiber intake was lower in this group but did not reach statistical significance. Diabetes mellitus, vaginal delivery, and history of recurrent vaginitis were more frequent among treatment-resistant patients. Multivariate analysis identified diabetes (*p* = 0.02), history of vaginal delivery (*p* = 0.01), and recurrent vaginitis (*p* = 0.009) as significant predictors of treatment failure.

**Conclusion:**

This study demonstrates that poor dietary quality, increased central adiposity, and metabolic and obstetric risk factors are associated with vaginitis treatment failure.

## Introduction

Vaginitis is a common and often recurrent condition characterized by inflammation or infection of the vaginal mucosa, typically presenting with symptoms such as pruritus, burning, abnormal discharge, dyspareunia, and malodor. Bacterial vaginosis (BV), vulvovaginal candidiasis (VVC), and Trichomonas vaginalis (TV) represent the most prevalent infectious causes, particularly among women of reproductive age (1, 2). Recent epidemiological evidence from population-based cohorts indicates that BV accounts for approximately 13.7% of diagnosed vaginal infections, followed by VVC (11.2%) and TV (0.7%) (2). These infections are frequently recurrent; in some populations, recurrence occurs in over 40% of cases within a year, contributing to both persistent symptoms and increased risk of reproductive complications including pelvic inflammatory disease, infertility, and preterm birth (3, 4). The pathophysiology of vaginitis is closely tied to disturbances in the vaginal microbiota. A eubiotic vaginal ecosystem, dominated by Lactobacillus species, maintains a low pH environment through lactic acid production, inhibits pathogenic growth, and strengthens mucosal immunity (5, 6). Disruption of this balance (dysbiosis) has been associated with increased susceptibility to BV, VVC, and STIs (7, 8). Despite this natural defense, vaginal infections occur repeatedly in many women (5). Emerging literature highlights dietary intake and metabolic status as underappreciated modulators of vaginal microbiota and infection outcomes. Diets high in refined sugars, saturated fats, and ultra-processed foods have been shown to increase the odds of BV, while those rich in fiber, antioxidants, and phytochemicals appear protective (9, 10). For instance, women with high dietary inflammatory index (DII) scores showed significantly greater odds of BV, particularly when coupled with low fiber intake (9). Micronutrient deficiencies such as folate and vitamin E, both known for their immunomodulatory and anti-inflammatory roles, have also been inversely associated with BV risk (11). Similarly, experimental models demonstrate that diets low in fiber reduce colonization resistance, promoting pathogenic overgrowth and prolonging infection (8). In addition to nutritional determinants, body composition – especially central adiposity – has emerged as a significant factor in vaginitis recurrence. Obesity-related metabolic inflammation, insulin resistance, and altered estrogen metabolism may interfere with immune and microbial homeostasis, increasing susceptibility and delaying recovery (12). According to data cited by Evans et al. vaginitis accounts for over 10 million office visits and more than $1.3 billion in healthcare expenditures annually (4). Given the clinical recurrence, emerging dietary and metabolic risk factors, and economic burden of vaginitis, a comprehensive approach to identify predictors of treatment failure is warranted.

### Objective and hypothesis

This study aims to investigate the association between anthropometric characteristics, dietary patterns, and gynecological history and the risk of treatment failure in women diagnosed with vaginitis. Specifically, it seeks to determine whether factors such as abdominal fat ratio, carbohydrate and sugar intake, presence of diabetes, and vaginal birth history contribute to persistent or recurrent infections despite medical treatment.

The main hypothesis of this study is that women with higher abdominal fat percentage, increased dietary intake of carbohydrates and simple sugars, and a history of metabolic or obstetric risk factors (e.g., diabetes, vaginal birth, prior vaginitis) have a higher likelihood of vaginitis treatment failure.

**The sub-hypotheses are as follows:**

H1: Higher abdominal fat percentage is associated with an increased risk of persistent vaginitis.

H2: Increased dietary intake of carbohydrates and simple sugars negatively affects treatment outcomes.

H3: Presence of diabetes mellitus significantly increases the likelihood of treatment failure.

H4: Women with a history of vaginal birth and recurrent vaginitis are more likely to experience treatment-resistant infections.

## Materials and methods

### Patient selection

This prospective observational study included women diagnosed with vaginitis at the Gynecology and Obstetrics Clinic. Patients planned for medical treatment, who returned for a follow-up examination, had their treatment response evaluated, and had complete data available were included in the study. Patients with co-existing gynecological conditions, irregular treatment adherence, or those who did not attend follow-up within the recommended time were excluded. The study was conducted in accordance with the Declaration of Helsinki. Ethical approval was obtained from the Scientific Research and Publication Ethics Committee of Osmaniye Korkut Ata University (Decision No: 2022/4/13). All participants provided written informed consent. The study was registered at ClinicalTrials.gov (NCT05882851).

### Study design

All patients received medical treatment as part of routine clinical care, in accordance with current gynecological guidelines. The treatment protocol was not determined by the research team. Follow-up evaluations were conducted 2 weeks later. Based on the independent physician’s clinical judgment at the follow-up visit, participants were categorized into two groups: those considered to have recovered (Group 1) and those with persistent symptoms (Group 2).

### Data collection and measurements

Demographic characteristics such as age, menopausal status, and presence of diabetes mellitus were recorded. Clinical history included previous episodes of vaginitis, history of childbirth, and delivery mode (vaginal or cesarean section). Anthropometric measurements were obtained by recording height and weight using standardized procedures, and body mass index (BMI) was calculated as weight in kilograms divided by height in meters squared (kg/m^2^). Body composition, including total body fat percentage and abdominal fat ratio, was measured using a TANITA MC series device based on multi-frequency bioelectrical impedance analysis (BIA). At the follow-up visit, dietary intake was assessed through a 3-day dietary recall. Nutritional analysis was performed using the BeBiS (Nutrition Information System) software. The proportions of carbohydrate and simple sugar intake, as well as the daily amount of fiber (grams), were calculated and recorded.

### Statistical analysis

Descriptive statistics including mean, standard deviation, minimum, and maximum values were calculated for continuous variables. Categorical variables were expressed as frequency and percentage. The independent samples *t*-test was used to compare means between the two groups. The Chi-square test was applied to evaluate relationships between categorical variables. Logistic regression analysis was performed using variables found to be statistically significant in univariate analysis. Backward and enter methods were used in model construction. A *p*-value of <0.05 was considered statistically significant. Analyses were conducted using MedCalc software and the e-picos platform.

## Results

**FIGURE 1:**
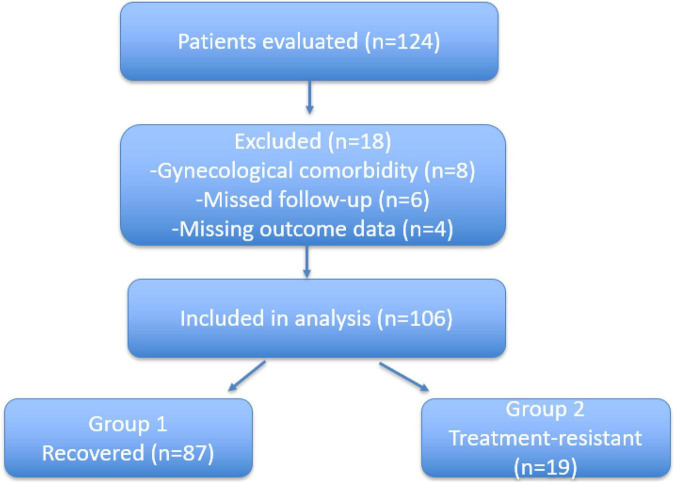
Flow diagram of patient selection and exclusion process.

Out of 124 patients initially assessed for eligibility, 18 were excluded due to predefined criteria: 8 had co-existing gynecological conditions, 6 failed to attend the scheduled follow-up visit, and 4 had incomplete clinical outcome data. Thus, the final sample consisted of 106 women diagnosed with vaginitis and followed through treatment response evaluation (Figure 1).

**FIGURE 2:**
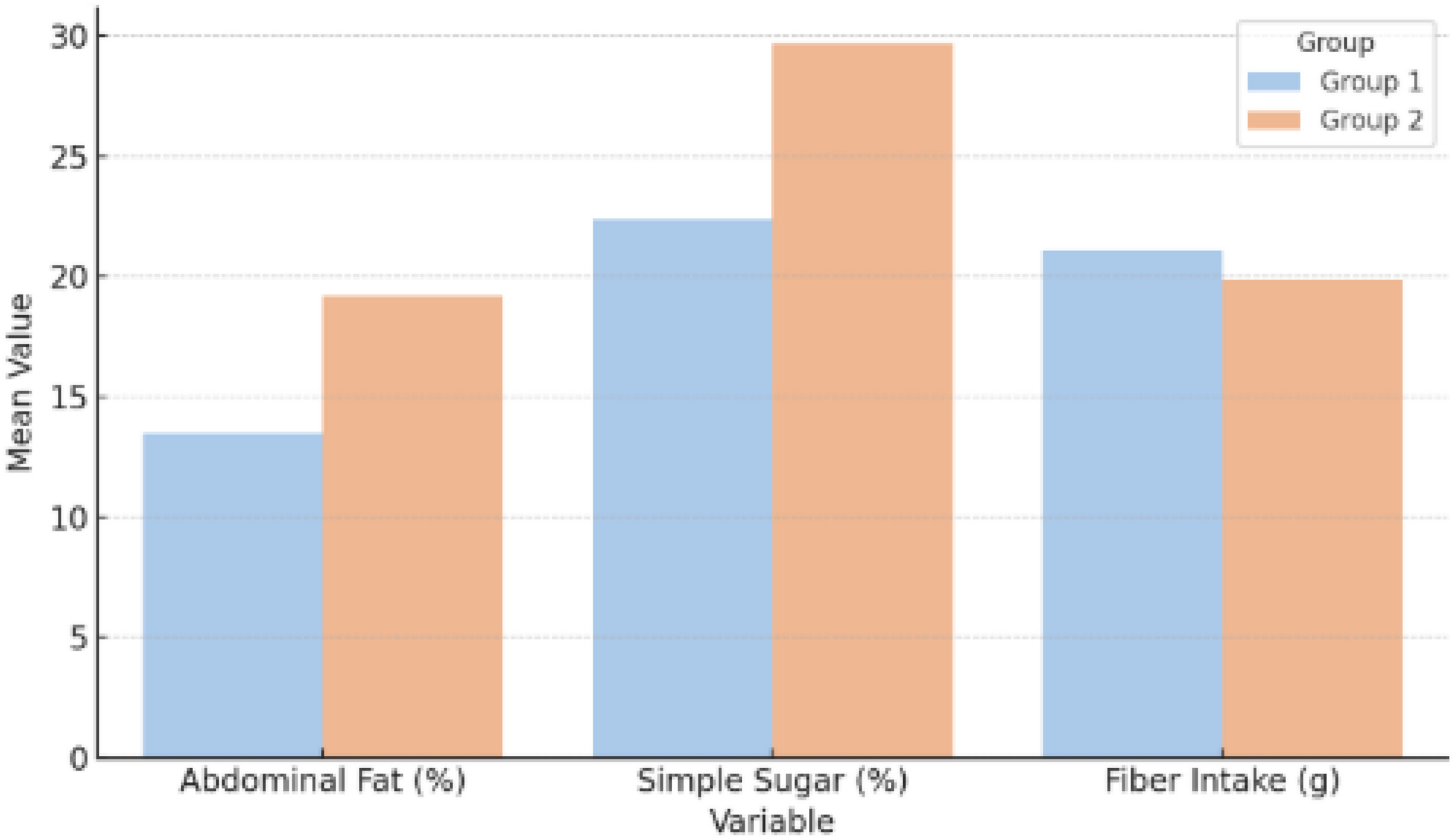
Comparative bar chart of abdominal fat %, simple sugar %, and fiber intake across groups.

Based on post-treatment clinical assessment, 87 patients (82.1%) were classified as recovered (Group 1), and 19 patients (17.9%) as treatment-resistant (Group 2). The mean age of participants was 45.05 ± 13.87 years. Comparisons of continuous variables between the two groups revealed that age, body mass index (BMI), total body fat percentage, and abdominal fat ratio were significantly higher in the treatment-resistant group. Likewise, the proportions of dietary carbohydrate intake and simple sugars were significantly elevated in Group 2 compared to Group 1 (*p* < 0.05 for all variables). Although mean fiber intake was slightly lower in Group 2, this difference did not reach statistical significance (*p* = 0.13). These findings are presented in Table 1 and visualized in Figure 2.

**TABLE 1:** Demographic, anthropometric, and nutritional characteristics of patients by group.

Variables	All patient (*n* = 106)	Group 1 (*n* = 87)	Group 2 (*n* = 19)	*p*-value
	***x* ± SD**	***x* ± SD**	***x* ± SD**	
Age (year)	45.05 ± 13.87	43.29 ± 10.59	49.12 ± 11.78	0.02*
BMI (kg/m^2^)	27.46 ± 7.52	26.15 ± 4.23	29.81 ± 6.99	0.008*
Total body fat (%)	35.25 ± 7.11	34.71 ± 4.83	37.92 ± 6.43	<0.001*
Abdominal fat (%)	36.29 ± 10.12	33.18 ± 7.46	41.28 ± 9.56	<0.001*
Carbohydrate intake (%)	72.46 ± 16.71	69.35 ± 8.91	76.41 ± 13.43	0.003*
Simple sugar (%)	12.42 ± 6.83	11.17 ± 4.61	14.02 ± 5.19	0.006*
Fiber intake (g)	20.73 ± 7.12	21.09 ± 5.12	19.87 ± 6.45	0.13*

*Independent *t*-test.

In terms of categorical characteristics, diabetes mellitus was more prevalent in the treatment-resistant group (63.2%) compared to the recovered group (33.3%), and the difference was statistically significant (*p* = 0.02). Similarly, the rates of previous childbirth (94.7% vs. 70.1%), history of vaginal delivery (88.9% vs. 57.3%), and prior episodes of recurrent vaginitis (94.7% vs. 64.4%) were markedly higher among patients in Group 2 (*p* = 0.03, *p* = 0.01, and *p* = 0.009, respectively). There were no significant differences between the groups regarding menopausal status or history of vaginal operations (*p* > 0.05 for both). These data are summarized in Table 2.

**TABLE 2:** Gynecological, endocrine, and obstetric characteristics.

	All patient (*n* = 106)	Group 1 (*n* = 87)	Group 2 (*n* = 19)	*p*-value*
	*n* (%)	*n* (%)	*n* (%)	
Menopausal period				
Premenopause	47 (44.3)	41 (47.1)	6 (31.6)	0.22
Postmenopause	59 (55.7)	46 (52.9)	13 (68.4)	
Diabetes mellitus				
Yes	41 (38.7)	29 (33.3)	12 (63.2)	0.02
No	65 (61.3)	58 (66.7)	7 (36.8)	
Vaginal operation history				
Yes	13 (12.2)	9 (10.3)	4 (21.5)	0.21
No	93 (87.8)	78 (89.7)	15 (78.5)	
Obstetric history				
Yes	79 (74.5)	61 (70.1)	18 (94.7)	0.03
No	27 (25.5)	26 (29.9)	1 (5.3)	
Mode of delivery				
Vaginal	51 (64.6)	35 (57.3)	16 (88.9)	0.01
Cesarean section	28 (35.4)	26 (42.7)	2 (11.1)	
Recurrent vaginitis				
Yes	74 (69.8)	56 (64.4)	18 (94.7)	0.009
No	32 (30.2)	31 (35.6)	1 (5.3)	

*Chi-square test.

**FIGURE 3:**
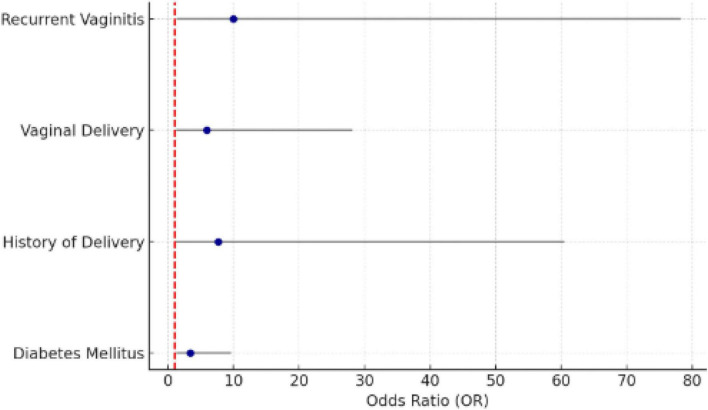
Forest plot showing odds ratios (OR) and 95% confidence intervals of multivariate predictors of treatment failure.

Multivariate logistic regression analysis was performed to identify independent predictors of treatment failure. The analysis demonstrated that diabetes mellitus (OR: 3.43, 95% CI: 1.22–9.64), a history of childbirth (OR: 7.67, 95% CI: 0.97–60.52), vaginal delivery (OR: 5.94, 95% CI: 1.26–28.14), and a history of recurrent vaginitis (OR: 9.96, 95% CI: 1.27–78.25) were significantly associated with increased risk of Treatment failure (*p* < 0.05 for all). Variables such as menopausal status and previous vaginal surgery were not significant in the multivariate model. These results are detailed in Table 3 and illustrated in Figure 3.

**TABLE 3:** Independent predictors of treatment failure.

	Odds ratio	95% Confidence interval (Lower–Upper)	*p*-value
Menopausal period	1.93	0.67–5.55	>0.05
Diabetes mellitus	3.43	1.22–9.64	<0.05
Vaginal operation history	2.31	0.63–8.49	>0.05
Obstetric history	7.67	0.97–60.52	<0.05
Vaginal birth	5.94	1.26–28.14	<0.05
Recurrent vaginitis	9.96	1.27–78.25	<0.05

Odds ratio (*p* < 0.05).

## Discussion

This study aimed to investigate whether anthropometric characteristics, dietary composition, and obstetric history are associated with treatment failure among women diagnosed with vaginitis. Our primary hypothesis was that higher abdominal fat percentage, elevated carbohydrate and simple sugar intake, and presence of diabetes mellitus or a history of vaginal delivery and recurrent vaginitis would increase the risk of Treatment failure. Our findings support this hypothesis and provide additional insight into modifiable contributors to treatment outcomes.

Patients in the treatment-resistant group exhibited significantly higher body mass index, abdominal fat ratio, and higher dietary intake of carbohydrates and simple sugars. In contrast, fiber intake was lower in this group. These findings align with earlier studies indicating that poor dietary quality – particularly high glycemic load and low fiber – may negatively affect the vaginal ecosystem and mucosal immunity (13, 14). High-glycemic diets have been associated not only with the prevalence of bacterial vaginosis but also with increased recurrence risk (15). The biological mechanisms underlying these associations include postprandial hyperglycemia, increased oxidative stress, and low-grade inflammation – all of which compromise epithelial defenses and alter vaginal pH, thus facilitating colonization by pathogenic microorganisms (16–18). In support of our findings, Mehrabani et al. demonstrated that refined carbohydrate consumption and reduced dietary fiber intake were significant dietary predictors of bacterial vaginosis and vaginal dysbiosis recurrence (19).

Our results also contribute to a growing body of literature highlighting the protective role of dietary fiber. Higher fiber consumption has been shown to promote Lactobacillus dominance in the vaginal microbiota, a critical factor for vaginal homeostasis (14). In a murine model, Megli et al. found that diets low in fiber and high in fat delayed the clearance of vaginal Group B Streptococcus colonization, while fiber-rich diets improved microbial resilience and host immune function. These findings suggest that fiber not only supports healthy microbiota but may also enhance treatment efficacy by facilitating infection clearance (8, 20).

Another key factor identified in our study was diabetes mellitus, which was significantly more prevalent in the treatment-resistant group. Hyperglycemia can alter glycogen levels in the vaginal epithelium, impair mucosal immunity, and reduce Lactobacillus colonization (21). This supports previous findings that diabetic women are at increased risk of recurrent vulvovaginal infections (22, 23). The pro-inflammatory state and microbiota shifts observed in diabetes may further explain the observed treatment failure (24). While menopause has been associated with decreased estrogen and glycogen levels – affecting microbial balance and epithelial integrity – our findings were consistent with studies suggesting that menopausal status alone does not significantly influence treatment success (5, 25). Although not directly assessed in our study, behavioral and sociodemographic risk factors such as vaginal douching, smoking, and sexual practices are well-established contributors to vaginal dysbiosis (17, 25). Given that these factors can modulate microbiota composition, they may also indirectly influence treatment outcomes and should be considered in future research.

In multivariate analysis, we identified diabetes mellitus, history of childbirth, vaginal delivery, and recurrent vaginitis as significant predictors of treatment failure. These results emphasize the importance of comprehensive risk assessment and lifestyle modification in managing vaginitis. Notably, obstetric history – particularly vaginal delivery – may lead to lasting changes in vaginal anatomy and microbial colonization patterns that predispose to recurrent infections. From a clinical standpoint, these findings suggest that dietary assessment and counseling should be incorporated into the routine management of women with vaginitis, especially those with a history of recurrence or metabolic risk factors. Nutritional interventions targeting carbohydrate reduction and fiber intake optimization may serve as valuable adjuncts to antimicrobial therapy.

This study has several limitations. It was prospective in design and conducted in a single-center setting, limiting generalizability. Dietary data were based on a 3-day recall, which may not capture habitual intake. *A priori* power analysis was not performed due to the unknown frequency of treatment failure in the target population. In conclusion, vaginitis treatment failure is significantly associated with poor nutritional quality, excess adiposity, diabetes mellitus, and specific obstetric risk factors. Incorporating dietary and metabolic screening into gynecological care may enhance therapeutic outcomes. Future prospective studies evaluating the effects of nutritional interventions on treatment success and microbial recovery are warranted.

## Conclusion

This study demonstrates that specific nutritional and metabolic factors – including high abdominal fat ratio, elevated dietary intake of carbohydrates and simple sugars, and the presence of diabetes mellitus – are significantly associated with vaginitis treatment failure. Additionally, a history of vaginal delivery and recurrent vaginitis emerged as notable obstetric predictors of poor treatment outcomes. These findings highlight the potential importance of integrating nutritional assessment, metabolic risk screening, and individualized dietary counseling into the clinical management of vaginitis. Future prospective, multi-center studies are warranted to evaluate whether modifying these risk factors can improve treatment response and reduce recurrence rates.

## Data Availability

The raw data supporting the conclusions of this article will be made available by the authors, without undue reservation.
